# A Comparative Study on a Novel Fibula Malleolus Cap to Increase the Accuracy of Oncologic Jaw Reconstruction

**DOI:** 10.3389/fonc.2021.743389

**Published:** 2022-01-05

**Authors:** Jingya Jane Pu, Wing Shan Choi, Wai Kan Yeung, Wei-Fa Yang, Wang-Yong Zhu, Yu-Xiong Su

**Affiliations:** ^1^ Division of Oral and Maxillofacial Surgery, Faculty of Dentistry, The University of Hong Kong, Hong Kong, Hong Kong SAR, China; ^2^ Division of Applied Oral Sciences & Community Dental Care, Faculty of Dentistry, University of Hong Kong, Hong Kong, Hong Kong SAR, China

**Keywords:** oncologic reconstruction, fibula free flap, head and neck cancer, jaw reconstruction, simultaneous dental implant, computer-assisted surgery (CAS), virtual surgical planning (VSP)

## Abstract

**Objectives:**

Although computer-assisted surgery using fibula flap has been widely applied for oncologic jaw reconstruction in recent years, the inaccurate positioning of the fibula harvest guide brings sliding and rotational errors, which leads to compromised accuracy in simultaneous implant placement and dental rehabilitation. This study aimed to develop a novel three-dimensional (3D)-printed patient-specific fibula malleolus cap to increase oncologic reconstruction accuracy.

**Methods:**

In this prospective comparative study with a recent historical control cohort, patients in need of oncologic jaw reconstruction with fibula free flaps were recruited. In the study group, the fibula was harvested with the guide of the malleolus cap, whereas in the control group, without the malleolus cap. Deviations of location and angulation of distal fibula osteotomies, jaw reconstruction segments, and simultaneous dental implants were compared.

**Results:**

Twenty patients were recruited, with 10 in each arm. The application of the malleolus cap significantly reduced the deviations in locations and angles of distal fibula osteotomies, from 9.5 to 4.1 mm and 25.3° to 8.7°. For the simultaneous dental implants placed in the fibula flaps, there was a significant increase in the accuracy of implant platform locations (the average deviation from 3.2 to 1.3 mm), apex locations (from 3.8 to 1.5 mm), and angles (from 11.3° to 4.6°). No significant difference was detected in the accuracy of fibula reconstruction segments.

**Conclusions:**

We developed a novel fibula malleolus cap to overcome the sliding and rotational errors during fibula flap harvesting for oncologic jaw reconstruction, with increased accuracy in simultaneous dental implants. This is a step forward to achieve a satisfactory functional outcome of jaw reconstruction with dental rehabilitation.

## 1 Introduction

Computer-assisted surgery (CAS) and three-dimensional (3D) printing have revolutionized head and neck oncologic reconstruction (1–5). Our serial studies on CAS and 3D printing facilitated a paradigm shift in jaw reconstruction, leading to a new era of “digitalization and precision surgery” (6–8). However, even with recent technology, the discrepancy between the surgical outcome and preoperative planning was 3.1 ± 1.4 mm (9). The recent publication by Zavattero et al. (10) reported the osseous accuracy ranging from 0.5 to 3 mm using patient-specific computer-aided design and computer-aided manufacturing (CAD-CAM) plates. Previous reports established the protocols for free flap reconstruction of jaws with simultaneous dental implant insertion, making the jaw-in-a-day technique the new state of the art (11–13). This required an even higher level of precision in planning and execution, as the deviation in osseous segments can be further amplified in the error in location and angulation of simultaneous dental implants, compromising the functional jaw reconstruction with dental rehabilitation. How to improve reconstruction accuracy to facilitate the accurate functional oncologic jaw reconstruction and dental rehabilitation is the last piece of the puzzle in computer-assisted jaw reconstruction.

**Figure 1:**
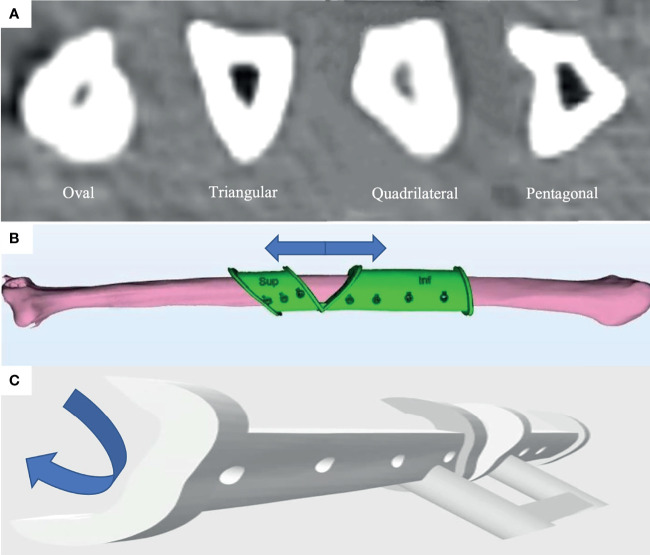
The sliding and rotational error occurred in fitting of fibula harvest guide will lead to inaccuracy in the reconstruction due to the different geometric shapes of the fibula in cross-section along its length. **(A)** Different geometric shapes of fibula (oval, triangular, quadrilateral, and pentagonal) of the fibula in cross-section along its length from the CT scan of a patient. **(B)** Illustration of the sliding error in the axial direction. **(C)** Illustration of the rotational error in cross-sectional direction.

Fibula free flap is the most commonly used reconstruction method for bony defects of jaws. Although fibula looks like a uniformly shaped long bone, the geometric shape of its cross-sectional anatomy actually differs a lot along its axis (**Figure 1A**). Thus, the accurate positioning of the fibula harvest guide is of premier importance to achieve the desired virtual surgical plan (VSP) in real surgery. However, it is well known that the fit for fibula harvest guides is less than ideal (14). In fact, in order to avoid undercuts that might prevent adaptation of guide to the fibula surface and protect the vessel pedicle on the medial surface of the fibula, only the geometry of the lateral surface can be used as a reference when designing the fibula harvest guide (**Figures 1B, C**). This leads to sliding and rotational errors when positioning the fibula harvesting guide. The traditional approach is to measure the distance from the skin marking over the lateral malleolus to locate the fibula harvesting guide. However, this approach brings inaccuracy. The lateral malleolus is a rounded 3D structure. The surgeon might take different reference points in the virtual surgical planning. Besides, the movable and sometimes distorted soft tissue will prevent the reliable and reproducible positioning of the ruler when the measurement is done intraoperatively. In addition to the longitudinal sliding error, the harvest guide may also rotate around the long axis of the fibula due to the relatively smooth rounded lateral surface of fibula especially when covered with periosteum and a thin cuff of muscle. Therefore, a method to reduce the sliding and rotational errors of the fibula harvest guide is urgently needed to increase the accuracy of jaw reconstruction.

Therefore, we developed a novel 3D-printed malleolus cap for fibula flap harvest and performed a comparative study to evaluate its effectiveness in enhancing the accuracy of fibula osteotomy, jaw reconstruction, and simultaneous dental implant placement.

## 2 Materials and Methods

### 2.1 Study Design

This was a two-arm clinical comparative study to evaluate the effectiveness of the malleolus cap design. The presurgical treatment plan, virtual surgery design, and surgical procedures were standardized in both arms. The single independent variable was whether a 3D-printed patient-specific fibula malleolus cap was applied to accurately locate the harvest guide intraoperatively.

### 2.2 Patient Recruitment

Patient selection criteria were consistent for both groups under the 3DJP16 clinical study (ClinicalTrials.gov Identifier: NCT03057223). Briefly, patients with oral and maxillofacial benign or malignant tumors or osteoradionecrosis who needed jaw resection and fibula flap reconstruction with 3D-printed patient-specific titanium plates were recruited. All patients were operated on by the same chief surgeon in a single center. Ten consecutive patients were prospectively recruited to the study group from June 2020 to December 2020, and 10 consecutive patients operated on from June 2019 to June 2020 were retrospectively recruited as the historical control group. This study was approved by the institutional review board of the University of Hong Kong Hospital Authority Hong Kong West Cluster (UW 16-315) with the informed consent signed.

### 2.3 Preoperative Virtual Surgical Planning

The workflow of our team in computer-assisted jaw reconstruction with 3D-printed patient-specific titanium implants was reported by Yang et al. (15). CT data were acquired and segmented to construct the 3D model of the donor fibula and the recipient jaw. Virtual reconstructive surgeries were conducted using ProPlan CMF 2.0 software (Materialise, Leuven, Belgium). Positions of simultaneous dental implants were determined in the prosthetically driven approach. Patient-specific fibula harvesting guides were designed using 3-Matic 13.0 (Materialise).

### 2.4 Design of the Malleolus Cap

**Figure 2:**
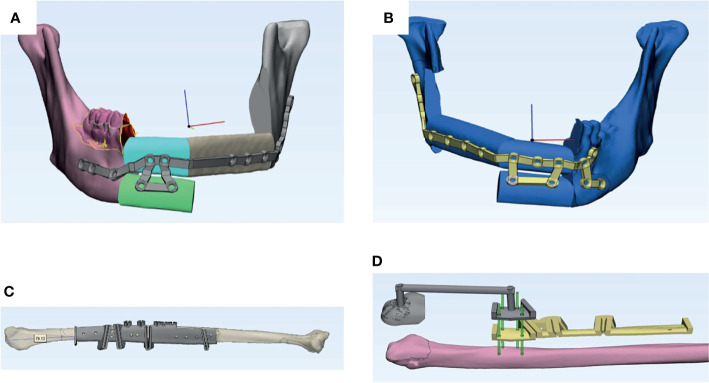
Two cases of fibula flap harvest using conventional measuring method vs. malleolus cap method. **(A)** Virtual surgical planning for a three-segment fibula flap in the control group. **(B)** Virtual surgical planning for a three-segment fibula flap in the study group. **(C)** Fibula harvest guide in the control group. Location of the guide intraoperatively depends on the measurement from the distal end of the guide to the lateral malleolus. **(D)** Two guides used in the study group. Yellow: Segmentation guide in the study group with a similar design as the fibula guide in the control group. Gray: Fibula guide for the distal osteotomy with malleolus cap design. Green: Rods showing the corresponding screw holes on the two guides in the study group.

The lateral malleolus is a tilted pyramid structure with the most prominent point located posteroinferiorly, which makes its lateral surface slant anteromedially. As illustrated in **Figure 2**, the lateral malleolus cap was designed as a 3-mm-thick cap that fits the surface morphology of the specific patient. The inner surface of the cap was relieved by 0.5 mm for the compressible skin covering the bony malleolus when under finger pressure. A distal stopper of 5 mm in length was added inferior to the posteroinferior end of the fibula to prevent the axial sliding error. The cap was connected to the routine harvest guide using 1-cm-diameter rigid connecting bars. We used a malleolus cap to locate the conventional fibula segmentation guide in a predetermined position by using the same fixation screw holes (**Figure 2D**) . So, once the malleolus cap was fixed, the final position of the fibula segmentation guide was also determined. With this design, we can minimize the axial and rotational errors caused by inaccurate positioning of the fibula segmentation guide. The surgical guides were printed with ISO-certified biocompatible autoclavable MED610 resin (Stratasys Ltd., USA) or NextDent SG (Vertex Dental, Netherlands).

### 2.5 Surgical Techniques

During the surgery, the 3D-printed patient-specific cutting guides were fitted to the tumor resection sites, and the osteotomies were made according to the VSP (**Figure 2**).

In the study group, the malleolus cap was fitted onto the lateral malleolus with the manual pressure in a posteromedial direction to locate the cap anteroposteriorly. Then, the cap was pushed superiorly until the distal stopper tightly engaged the inferior end of the malleolus. The thin compressible soft tissue over the lateral malleolus allowed the correct fitting of the patient-specific malleolus cap under finger pressure. When the cutting guide was accurately located, the osteotomy guide fitted onto the fibula bone surface automatically and fixed with 8-mm screws that were inserted perpendicular to the surface of the bone to minimize the rotational error induced by the incorrect torque of the screws. In this way, the location of the fibula harvest guide was controlled in all three dimensions.

In the control group, the lateral malleolus was marked on the skin by palpation. After exposure of the fibula, the harvest guide was fitted to the estimated location by measuring the distance from the lateral malleolus skin marking to the osteotomy site according to the VSP.

**Figure 3:**
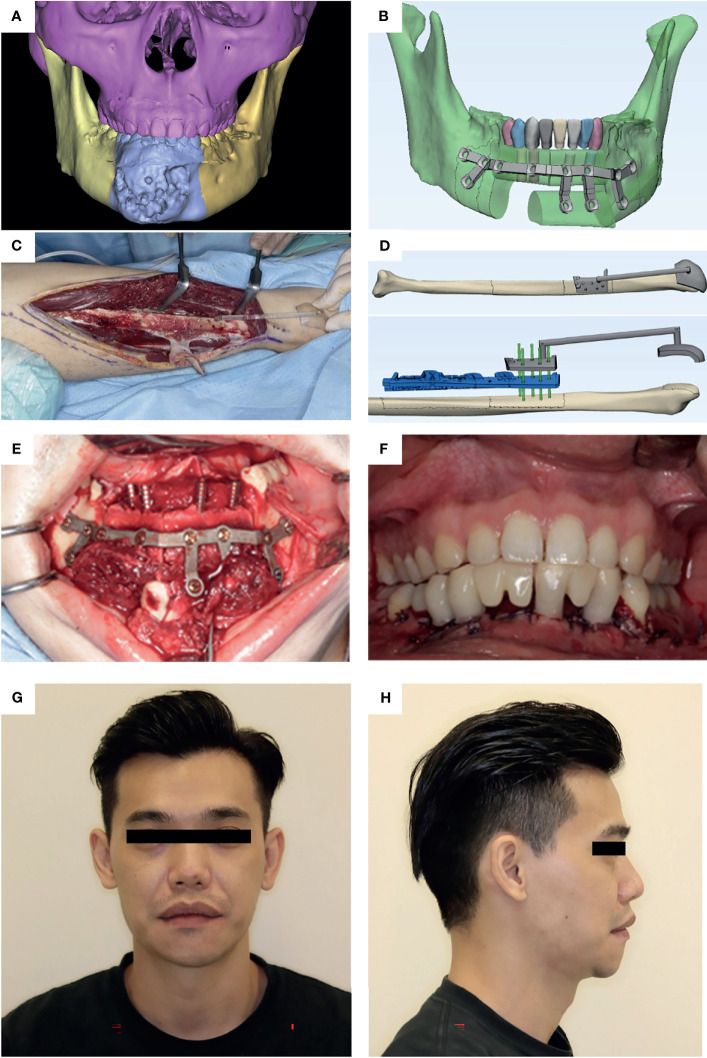
A case illustration of a 32-year-old male diagnosed with ossifying fibroma in the anterior mandible who received segmental mandibulectomy and reconstruction using fibula free flap harvested with lateral malleolus cap. **(A)** Preoperative CT image indicates the destructive mass in the anterior mandible. **(B)** The 3D-printed patient-specific surgical plate designed to fix bone segments with dental implants. **(C)** Harvest guide with malleolus cap applied in the surgery. **(D)** The fibula guides. Gray: Fibula harvest guide with malleolus cap for distal osteotomy cut. Blue: Segmentation and implant guide. Green: Rods showing the corresponding screw holes on the two guides. **(E)** The bone-plate complex is transferred to repair the defect site. **(F)** Intraoral image shows the accurate position of implants as planned. “Jaw-in-a-day” procedure was completed by immediate loading of dental implants with fixed dental bridges. An excellent occlusal relationship was achieved. **(G)** Postoperative photo (frontal view). **(H)** Postoperative photo (right profile view).

Distal fibula osteotomies were performed with the fibula harvest guides in both groups. Simultaneous dental implants were placed into the fibula before segmentation and division of the vessel pedicle. Fibula flap segments were transferred to repair the defect and fixed to the remaining jaw with 3D-printed patient-specific titanium plates. The fixation screw holes of the patient-specific titanium plates corresponded to the screw holes in the harvest guides of fibula and cutting guides of the recipient jaws (**Figure 3**).

### 2.6 Outcomes Assessment

**Figure 4:**
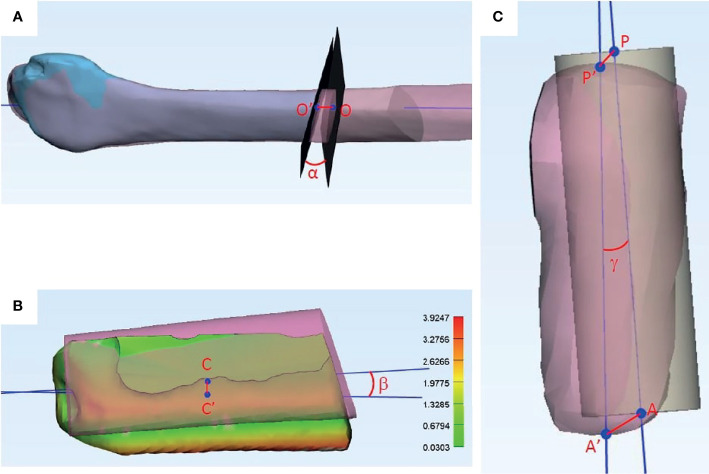
References used in accuracy analyses. **(A)** Fibula distal osteotomy accuracy analyses. Blue: Distal end of fibula after harvest. Pink: Fibula in preoperative virtual surgical planning. O: Center of the planned osteotomy plane. O’: Center of the actual osteotomy plane. O-O’: Axial deviation of the osteotomy plane. α: Angle deviation of the osteotomy plane. **(B)** Reconstruction segment accuracy analyses. Colored: Fibula segment at recipient site in postoperative CT scan. Pink: Fibula segment in preoperative virtual surgical planning. C: Center of the planned fibula segment. C’: Center of the actual fibula segment. C-C’: Center point deviation of the fibula segment. β: Angle deviation of the fibula segment. Absolute distance deviation represented in the color map. **(C)** Implant accuracy analyses. Yellow cylinder: Implant position in the virtual surgical plan. Pink cylinder: Actual implant position in the postoperative CT scan. P: Center point of implant platform in the virtual surgical plan. P’: Center point of implant platform in the actual implant. A: Apex of the implant in the virtual surgical plan. A’: Apex of actual implant placed. P-P’: Deviation in implant platform position (mm). A-A’: Deviation in implant apex position (mm). γ: Angle deviation of long axes of implants.

Spiral CT of the lower limbs and reconstructed jaws were acquired postoperatively. Based on the data of CT scan, 3D models of the distal end of the remaining fibula, reconstructed jaws, and simultaneous dental implants were built using ProPlan CMF 2.0 software (Materialise, Leuven, Belgium). The 3D models were imported to 3-Matic 13.0 (Materialise) for comparison of the corresponding items with the preoperative plan. The references used for analyses are illustrated in **Figure 4**.

#### 2.6.1 Fibula Osteotomy Accuracy Analyses

The postoperative fibula model was superimposed with the preoperative fibula using the best fit calculation embedded in the program. The real osteotomy plane was taken as the best plane that fits the osteotomy end of the distal remaining fibula. The angulation between this plane and the planned osteotomy plane was measured as the deviation in angulation of the osteotomy cut, representing the rotational error of distal fibula osteotomy. The long axis of the fibula was created and the intersection points between the long axis and the two osteotomy planes were taken to mark the location of the osteotomy planes. The distance between the intersection points generated by the real and planned osteotomy planes was measured as the axial deviation of the distal osteotomy, representing the sliding error of fibula harvesting.

#### 2.6.2 Mandible Reconstruction Analyses

**Figure 5:**
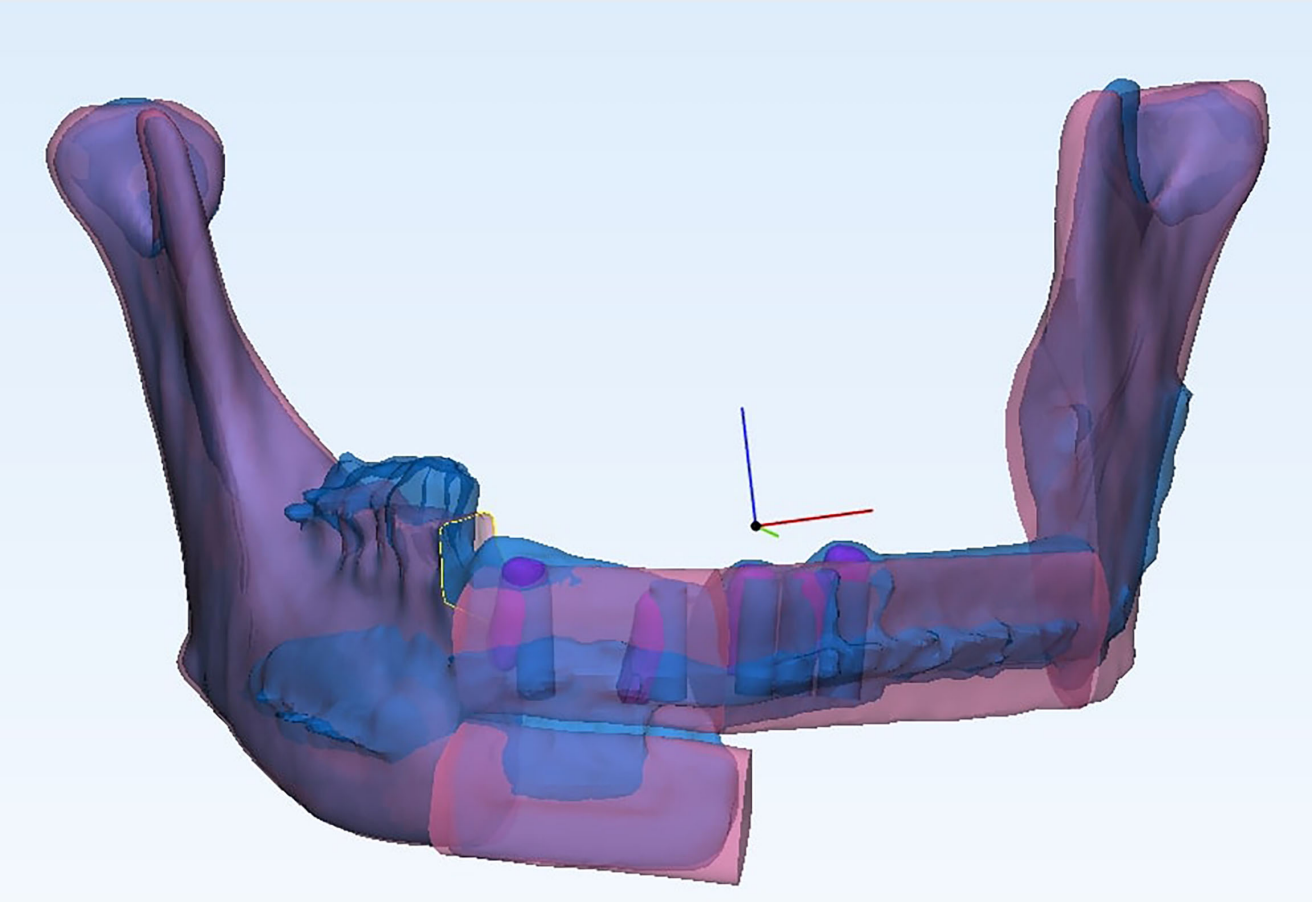
The postoperative reconstructed jaw was superimposed with the preoperative plan with the best fit of the non-operated part of the native mandible.

As described in our previous study, the postoperative reconstructed jaw was superimposed with the preoperative plan with the best fit of the non-operated part of native mandible (**Figure 5**) (9). The absolute distance deviation between the surfaces of reconstruction segments postoperatively and the preoperative plan was calculated and represented by a hot map. The distance between two center points was measured to represent the spatial deviation. The long axis of each segment was generated and compared to measure the angle deviation from the plan.

#### 2.6.3 Simultaneous Dental Implant Accuracy Analyses

Similar to our published methodology, the actual location of implants inserted was represented as the 3D models built from the postoperative CT scan (16). Long axes of the actual and the planned dental implants were compared to demonstrate the angle deviation in implant insertion. The intersection points of the long axis with the top and bottom of the dental implant were taken as the center of the platform and the implant apex. Deviation in platform and apex locations was measured to assess the accuracy of implant location.

### 2.7 Statistical Analysis

All statistical analyses were performed using IBM SPSS Statistics Version 25. Categorical data were presented as counts with proportions and compared using chi-square test or Fisher’s exact test where appropriate. The normality of continuous data was tested using Shapiro–Wilk’s test (p > 0.05). Descriptive statistical analyses were performed with results presented by mean with standard deviation (SD) for normally distributed data and medians with interquartile range (IQR) for skewed data. Independent-samples *t*-test and Mann–Whitney *U* test were used to compare the difference between the two groups for normally distributed data and skewed data, respectively. All tests and reported *p* values were two-sided. A *p* value of <0.05 was considered statistically significant.

## 3 Results

### 3.1 Patient Demographic Background

Twenty patients who underwent jaw reconstructions with fibula free flaps and 3D-printed patient-specific titanium plates were included in the study, with 10 patients in each arm. All 20 patients were of Han Chinese ethnicity. All 20 fibula free flaps survived. The postoperative follow-up rate was 100%. A total of 13 and 18 simultaneous dental implants were inserted in the study and control groups, respectively. The demographic data and reconstruction characteristics are presented in **Table 1**. No significant difference was detected between the two groups.

**Table 1:** Patient demographics and reconstruction characteristics.

	Study (n = 10)	Control (n = 10)	*p* value
**Gender**			
Male	6	4	0.66
Female	4	6	
**Age (mean)**	53	60	0.44
**Diagnosis**			0.80
SCC	5	7	
Other malignancy	1	1	
Benign jaw lesions	2	2	
Osteoradionecrosis	2	0	
**Reconstruction site**			
Maxilla	2	2	1.00
Mandible	8	8	
**Staging**			
pT1/2	0	3	0.31
pT3/4	6	5	
NA	4	2	
**Fibula segments (mean)**	2	2	0.73
**Fibula segment length (mm)**	48.2	47.3	0.90
**Implants (total)**	13	18	0.42

### 3.2 Outcomes Analyses

The accuracy of distal osteotomies of the fibulas, reconstruction segments, and implants was analyzed for all 20 cases and compared between the study and the control groups. The postoperative measurements for accuracy analyses were performed by two independent assessors blinded from the grouping. The inter-assessor agreement was good to excellent. The average values of the two assessors were taken for the final analyses. The results are presented in **Table 2**.

**Table 2:** Accuracy analyses results.

	Study (n = 10)	Control (n = 10)	*p* value	Mean difference	95% confidence interval
Fibula donor site analyses					
Distal fibula osteotomy					
- Axial deviation (mm)	4.1 ± 2.7	9.5 ± 6.3	0.02*	-5.5	-0.9 to -10.0
- Angle deviation (degrees)	8.7 ± 5.0	25.3 ± 13.1	<0.01**	-16.6	-6.9 to -26.4
Reconstruction segment analyses					
Absolute distance deviation	1.5 ± 0.8	1.9 ± 0.7	0.23		
Angle deviation	5.2 ± 2.5	5.8 ± 2.8	0.63		
Center points	2.1 ± 1.8	3.9 ± 1.3	0.44		
Implant analyses					
Platform deviation	1.3 ± 0.8	3.2 ± 1.4	<0.01**	-1.8	-1.1 to -2.6
Apex deviation	1.5 ± 0.8	3.8 ± 1.3	<0.01**	-2.2	-1.4 to -3.1
Angle deviation	4.6 ± 1.7	11.3 ± 7.3	0.01*	-6.8	-3.1 to -10.5

*All p values lower than 0.05 are indicated with an asterisk (*).

**All p values lower than 0.01 are indicated with double asterisks (**).

#### 3.2.1 Fibula Donor Site Osteotomy Accuracy Analyses

The accuracy of the distal osteotomy of the fibula increased significantly in the malleolus cap group. The axial deviation in location of the osteotomy plane from the VSP decreased from 9.5 ± 6.3 mm to 4.1 ± 2.7 mm (mean difference = -5.5 mm, 95% CI = -0.9 to -10.0, *p* = 0.02). The deviation of the distal osteotomy angle decreased from 25.3° ± 13.1° (mean difference = -16.6°, 95% CI = -6.9 to -26.4, *p* < 0.01).

#### 3.2.2 Reconstruction Segment Analyses

Three-dimensionally printed patient-specific titanium plates were used in both groups. No significant difference in the accuracy of reconstruction segments was detected in terms of absolute distance deviation, reconstruction segment angle deviation, or reconstruction segment center point deviation.

#### 3.2.3 Implant Accuracy Analyses

There was a significant improvement in the accuracy of simultaneous dental implants in the malleolus cap group. The platform deviation from the VSP decreased from 3.2 ± 1.4 mm to 1.3 ± 0.8 mm (mean difference = -1.8 mm, 95% CI = -1.1 to -2.6, – < 0.01). The deviation of apical point of the implants decreased from 3.8 ± 1.3 mm to 1.5 ± 0.8 mm (mean difference = -2.2 mm, 95% CI = -1.4 to -3.1, p < 0.01). The angle deviation reduced to 4.6° ± 1.7° in the study group compared to 11.3° ± 7.3° in the control group (mean difference = -6.8°, 95% CI = -3.1° to -10.5°, *p* = 0.01).

## 4 Discussion

To the best of our knowledge, this is the first clinical study aiming to reduce the sliding and rotational errors of fibula flap harvest. We propose and develop a novel fibula malleolus cap, which has demonstrated the effectiveness in accurately guiding the location and angulation during fibula flap harvest in a clinical comparative study.

The difference in geometric shape of cross-sectional anatomy of fibula has been overlooked in fibula flap oncologic jaw reconstruction so far. When simultaneous dental implantation is planned, the changes in cross-sectional shapes (such as oval, triangle, quadrilateral, and pentagonal) (**Figure 1A**) can make a significant impact to the dental implant position and angulation. The sliding and rotational errors may lead to implant thread exposure. Rotational error will add to the problem with prosthetic rehabilitation due to wrong angulation. Traditionally, when harvesting the fibula free flap, the location of the fibula cutting guide was determined by the measurement from the lateral malleolus. This leads to sliding and rotational errors as shown in **Figures 1B, C**. In our study, the sliding error in the control group was as large as 9.5 mm. With the application of the malleolus cap design, the error was reduced by more than half to 4.1 mm. This sliding error will lead to different shapes of the fibula harvested compared to the preoperative plan. The rotational error was also a concern especially when the fibula was covered with the periosteum and a thin cuff of muscle when the fibula cutting guide was fitted. Our results showed that the malleolus cap could significantly reduce the rotational error and improve the accuracy of cutting angle from 25.3° to 8.7°.

Interestingly, our results showed no significant difference in the accuracy of reconstructive fibula segments with or without the use of malleolus cap. Although there is no consensus on the parameters to use for the analysis of jaw reconstruction accuracy, the relatively commonly used measurements such as the average deviation of the surface location, center point, and angulation were adopted in this study (17). The results were comparable to the studies by Schepers et al. (18) and De Maesschalck et al. (19). The accuracy of fibula segments in the reconstructed mandible is mostly determined by the mandible osteotomy guides and 3D-printed patient-specific titanium plate (9). This also explains the practice in some parts of the world where a generic fibula CT scan data are used for designing the fibula harvest guide when the CT scan for the specific patient is not available (20). The sliding and rotational errors in fibula harvest might not be clinically significant if simultaneous dental implants were not planned. Although, currently, there has been no widely accepted criteria for minimal clinically important difference (MCID) in jaw reconstruction, MCID will be different whether dental implant rehabilitation is to be performed or not. The application of simultaneous dental implants has further pushed the front of functional jaw reconstruction with dental rehabilitation. When simultaneous dental implants were planned, it required high accuracy of the fibula harvest guides to insert the implants into the designated position and angulation.

Of note, the accuracy of simultaneous implants was significantly increased in the fibula malleolus cap group. Theoretically, with perfect execution of the plan, the implant location, direction, and depth should all be guided. However, during the surgery, due to the sliding and rotational error in the fibula harvest guide in the control group, the segment of fibula used for the dental implants is not exactly the segment used in the preoperative planning. Considering the different shape and size of fibula at different locations (**Figure 1A**), the best fit for the implant guide is not at the planned position. Moreover, when the shape and angulation of the fibula are different from the one in the preoperative planning, there may be a problem of thread exposure at the platform level or overdrilling and exposure of implant apices. In these situations, surgeons may adjust the depth and less often the angle of the implants in the fibula intraoperatively to fully submerge the implants into the fibula. This further contributes to the inaccuracy in simultaneous dental implants in fibula free flap with bone-borne guides in the control group. Previous studies investigated various methods for improving the accuracy of simultaneous dental implants in jaw reconstruction. Zweifel et al. (21) reported the use of a tooth-borne or plate-borne implant position verification guide for improving the accuracy of dental implants. This method relied on the patient’s existing dentition or the accurate location of the patient-specific fixation plates. Schepers et al. (22) advocated the fabrication of an occlusal splint to assist in the fixation of fibula segments intraoperatively in order to obtain a satisfactory location for dental implant-supported prosthesis, which required accurate registration of jaw relation before the operation and the reproduction of a correct condyle position intraoperatively. Literature showed that the designs of implant positioning guides and methods of accuracy analyses varied, which made the comparison between studies difficult. In paper series by Schepers et al. (13, 22), they reported a center deviation of 5.5 mm and an angle deviation of 6.1° in their group of simultaneous dental implants in jaw reconstruction. With the application of the malleolus cap in the present study, implant platform and apex deviations were reduced to 1.3 and 1.5 mm, respectively, with an angular deviation of 4.6°. This accuracy level approached what we could achieve with the conventional guided dental implant placement directly into the native maxilla and mandible. This was consistent with the result by Zweifel et al. (21) in a comparative study that aimed to verify the use of a splint for verification of correct location and angulation of simultaneous dental implants. Meta-analysis by Tahmaseb et al. (23) reported a deviation of 1.2 mm at the entry point and 1.4 mm at the apex with an angle deviation of 3.5°. Another review by Zhou et al. (24) also yielded similar results of an average horizontal deviation of 1.25 mm at entry point and angulation deviation of 4.1° in guided dental implants. This proved that with careful presurgical planning and good intraoperative execution, the accuracy of simultaneous dental implants in fibula free flaps with bone-borne implant guides can be comparable to the dental implants placed directly into the native jaws.

There are certain limitations in our study that need to be addressed. The anatomy of fibula may vary among different races. The current study was based on Han Chinese population. Experience and data on different ethnicity groups of patients are still yet to be reported. Sample size calculation was not possible due to the lack of previous publication/data to estimate the power. With a total of 20 patients, our results reached statistical significance and served the purpose of proving the feasibility and effectiveness of this new innovation. A further randomized controlled clinical trial can be designed with an estimated sample size based on our results. A recent historical control group was adopted in our study. There was no randomization between the two groups. However, all the surgeries were performed by the same chief surgeon with the same design of surgical guides and patient-specific titanium plates except the difference of malleolus cap, and the difference of median date of surgery between the two groups was only 6.5 months, thus reducing the bias to a minimum. A prospective randomized clinical trial would be preferred to achieve a more persuasive conclusion. However, because of the significant improvement of the clinical outcomes, the novel malleolus cap design has become a routine practice for computer-assisted jaw reconstruction with simultaneous dental implants in our center, which makes a randomized trial less likely in the future.

This is the first study assessing the accuracy of the fibula harvest guide in guiding the location and angulation of the fibula osteotomy. The novel fibula malleolus cap developed by us can significantly increase the accuracy of the fibula osteotomy, thus making the dental prosthetic rehabilitation with simultaneous dental implants more precise, approaching a similar accuracy level of the guided implant surgeries in native maxilla and mandible. The results will push forward the frontiers of computer-assisted functional oncologic jaw reconstruction with dental rehabilitation.

## Data Availability Statement

The original contributions presented in the study are included in the article/**Supplementary Material**. Further inquiries can be directed to the corresponding author.

## Ethics Statement

The studies involving human participants were reviewed and approved by the institutional review board of the University of Hong Kong Hospital Authority Hong Kong West Cluster. The patients/participants provided their written informed consent to participate in this study. Written informed consent was obtained from the individual(s) for the publication of any potentially identifiable images or data included in this article.

## Author Contributions

JP contributed to study design and data acquisition and interpretation, performed all statistical analyses, and drafted the article. WC contributed to surgical planning, follow-up with the patients, and critical appraisal of the article. WY contributed to data collection and critically revised the article. W-FY contributed to statistical analyses and critical appraisal of the article. W-YZ contributed to surgical planning and critical appraisal of the article. Y-XS contributed to conception, design, and data interpretation and critically revised the article. All authors approved the final article for publication.

## Funding

The study was supported by the University Research Committee Platform Technology Funding, HKU Seed Fund for Translational and Applied Research (202010160032), Health and Medical Research Fund (Project no.: 08192096), Food and Health Bureau, Hong Kong, and Guangdong Science and Technology Department (No. 2019A050516001).

## Conflict of Interest

The authors declare that the research was conducted in the absence of any commercial or financial relationships that could be construed as a potential conflict of interest.

## Publisher’s Note

All claims expressed in this article are solely those of the authors and do not necessarily represent those of their affiliated organizations, or those of the publisher, the editors and the reviewers. Any product that may be evaluated in this article, or claim that may be made by its manufacturer, is not guaranteed or endorsed by the publisher.

## References

[1] Hirsch DL Garfein ES Christensen AM Weimer KA Saddeh PB Levine JP. Use of Computer-Aided Design and Computer-Aided Manufacturing to Produce Orthognathically Ideal Surgical Outcomes: A Paradigm Shift in Head and Neck Reconstruction. J Oral Maxillofac Surg (2009) 67(10):2115–22. doi: 10.1016/j.joms.2009.02.007 19761905

[2] Hanasono MM Skoracki RJ. Computer-Assisted Design and Rapid Prototype Modelling in Microvascular Mandible Reconstruction. Laryngoscope (2012) 123:597–604. doi: 10.1002/lary.23717 23007556

[3] Matros E Albornoz CR Rensberger M Weimer K Garfein ES. Computer-Assisted Design and Computer-Assisted Modeling Technique Optimization and Advantages Over Traditional Methods of Osseous Flap Reconstruction. J Reconstr Microsurg (2014) 30(5):289–96. doi: 10.1055/s-0033-1358789 24323480

[4] Hanasono MM Matros E Joseph D. Important Aspects of Head and Neck Reconstruction. Plast Reconstr Surg (2014) 134(6):968–80. doi: 10.1097/PRS.0000000000000722 25415120

[5] Rana M Chin SJ Muecke T Kesting M Groebe A Riecke B. Increasing the Accuracy of Mandibular Reconstruction With Free Fibula Flaps Using Functionalized Selective Laser-Melted Patient-Specific Implants: A Retrospective Multicenter Analysis. J Craniomaxillofac Surg (2017) 45(8):1212–19. doi: 10.1016/j.jcms.2017.04.003 28552201

[6] Pu JJ Choi WS Yu P Wong MCM Lo AWI Su YX. Do Predetermined Surgical Margins Compromise Oncological Safety in Computer-Assisted Head and Neck Reconstruction? Oral Oncol (2020) 111:104914. [published online ahead of print, 2020 Jul 22]. doi: 10.1016/j.oraloncology.2020.104914 32712577

[7] Yang WF Choi WS Leung YY Curtin JP Du R Zhang CY. Three-Dimensional Printing of Patient-Specific Surgical Plates in Head and Neck Reconstruction: A Prospective Pilot Study. Oral Oncol (2018) 78:31–6. doi: 10.1016/j.oraloncology.2018.01.005 29496055

[8] Powcharoen W Yang WF Li KY Zhu W Su YX. Computer-Assisted Versus Conventional Freehand Mandibular Reconstruction With Fibula Free Flap: A Systemic Review and Meta-Analyses. Plast Reconstr Surg (2019) 144(6):1417–28. doi: 10.1097/PRS.0000000000006261 31764662

[9] Yang WF Choi WS Wong MC Powcharoen W Zhu W Tsoi JKH. Three-Dimensionally Printed Patient-Specific Surgical Plates Increase Accuracy of Oncologic Head and Neck Reconstruction Versus Conventional Surgical Plates: A Comparative Study. Ann Surg Oncol (2021) 28(1):363–75. doi: 10.1245/s10434-020-08732-y PMC7752789 32572853

[10] Zavattero E Bolzoni A Dell’Aversana G Santagata M Massarelli O Ferri A. Accuracy of Fibula Reconstruction Using Patient-Specific Cad/Cam Plates: A Multicenter Study on 47 Patients. Laryngoscope (2021) 131(7):E2169–75. doi: 10.1002/lary.29379 33452834

[11] Zweifel D Bredell MG Essig H Gander T Lanzer M Rostetter C. Total Virtual Workflow in CAD-CAM Bony Reconstruction With a Single Step Free Fibular Graft and Immediate Dental Implants. Br J Oral Maxillofac Surg (2018) 56(9):859–63. doi: 10.1016/j.bjoms.2018.09.010 30293801

[12] Levine JP Bae JS Soares M Brecht LE Saadeh PB Ceradini DJ. Jaw in a Day: Total Maxillofacial Reconstruction Using Digital Technology. Plast Reconstr Surg (2013) 131(6):1386–91. doi: 10.1097/PRS.0b013e31828bd8d0 23714799

[13] Schepers RH Kraeima J Vissink A Lahoda LU Roodenburg JL Reintsema H. Accuracy of Secondary Maxillofacial Reconstruction With Prefabricated Fibula Grafts Using 3D Planning and Guided Reconstruction. J Craniomaxillofac Surg (2016) 44(4):392–9. doi: 10.1016/j.jcms.2015.12.008 26867807

[14] Wilde F Hanken H Probst F Scheramm A Heiland M Cornelius CP. Multicenter Study on the Use of Patient-Specific CAD/CAM Reconstruction Plates for Mandibular Reconstruction. Int J CARS (2015) 10:2035–51. doi: 10.1007/s11548-015-1193-2 25843949

[15] Yang WF Zhang CY Choi WS Zhu WY Li DTS Chen XS. A Novel ‘Surgeon-Dominated’ Approach to the Design of 3D-Printed Patient-Specific Surgical Plates in Mandibular Reconstruction: A Proof-of-Concept Study. Int J Oral Maxillofac Surg (2020) 49(1):13–21. doi: 10.1016/j.ijom.2019.05.005 31230767

[16] Zhu WY Su YX Pow EHN Yang WF Qin L Choi WS. “Three-In-One” Patient-Specific Surgical Guides for Simultaneous Dental Implants in Fibula Flap Jaw Reconstruction: A Prospective Case Series. Clin Implant Dent Relat Res (2020) 23:43–53. doi: 10.1111/cid.12954 33180980

[17] van Baar GJC Forouzanfar T Liberton N Winters HAH Leusink FKJ. Accuracy of Computer-Assisted Surgery in Mandibular Reconstruction: A Systematic Review. Oral Oncol (2018) 84:52–60. doi: 10.1016/j.oraloncology.2018.07.004 30115476

[18] Schepers RH Raghoebar GM Vissink A Stenekes MW Kraeima J Roodenburg JL. Accuracy of Fibula Reconstruction Using Patient-Specific CAD/CAM Reconstruction Plates and Dental Implants: A New Modality for Functional Reconstruction of Mandibular Defects. J Craniomaxillofac Surg (2015) 43:649–57. doi: 10.1016/j.jcms.2015.03.015 25911122

[19] De Maesschalck T Courvoisier DS Scolozzi P. Computer-Assisted Versus Traditional Freehand Technique in Fibular Free Flap Mandibular Reconstruction: A Morphological Comparative Study. Eur Arch Otorhinolaryngol (2017) 274:517–26. doi: 10.1007/s00405-016-4246-4 27501991

[20] Luu K Pakdel A Wang E Prisman E. In House Virtual Surgery and 3D Complex Head and Neck Reconstruction. J Otolaryngol Head Neck Surg (2018) 47(1):75. doi: 10.1186/s40463-018-0320-9 30541624 PMC6290522

[21] Zweifel D Bredell MG Lanzer M Rostetter C Rücker M Studer S. Precision of Simultaneous Guided Dental Implantation in Microvascular Fibular Flap Reconstructions With and Without Additional Guiding Splints. J Oral Maxillofac Surg (2019) 77(5):971–6. doi: 10.1016/j.joms.2018.12.025 30689969

[22] Schepers RH Raghoebar GM Vissink A Lahoda LU Van der Meer WJ Roodenburg JL. Fully 3-Dimensional Digitally Planned Reconstruction of a Mandible With a Free Vascularized Fibula and Immediate Placement of an Implant-Supported Prosthetic Construction. Head Neck (2013) 35(4):E109–14. doi: 10.1002/hed.21922 22025326

[23] Tahmaseb A Wa V Wismeijer D Coucke W Even C. The Accuracy of Static Computer-Aided Implant Surgery: A Systemic Review and Meta-Analysis. Clin Oral Implants Res (2018) 29(Suppl 16):416–35. doi: 10.1111/clr.13346 30328191

[24] Zhou W Liu Z Song L Kuo CL Shafer DM. Clinical Factors Affecting the Accuracy of Guided Implant Surgery—A Systematic Review and Meta-Analysis. J Evid Based Dent Pract (2018) 18:28. doi: 10.1016/j.jebdp.2017.07.007 29478680

