# Supplementary figures and images for: A Comparative Study on a Novel Fibula Malleolus Cap to Increase the Accuracy of Oncologic Jaw Reconstruction
Source: Front Oncol. 2022 Jan 5;11:743389. doi: 10.3389/fonc.2021.743389 (PMC8767155; doi:10.3389/fonc.2021.743389)

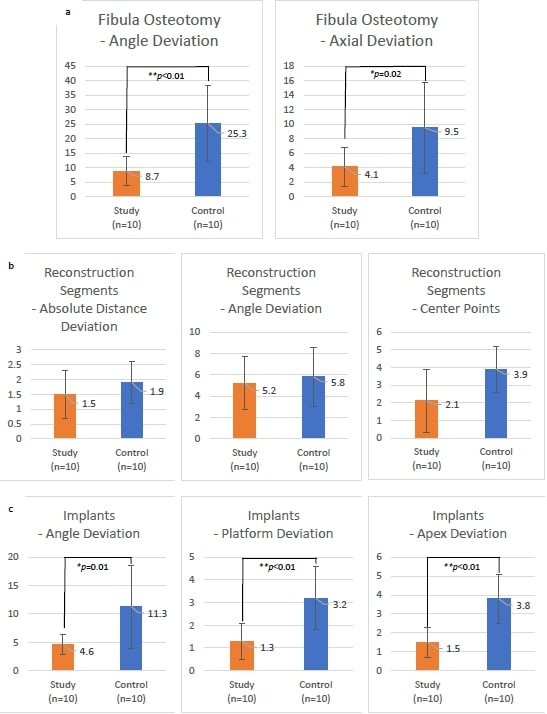

Supplement: Supplementary file 1 [file Image_1.jpeg]
